# Engagement in a Diabetes Self-management Website: Usage Patterns and Generalizability of Program Use

**DOI:** 10.2196/jmir.1391

**Published:** 2011-01-25

**Authors:** Russell E Glasgow, Steven M Christiansen, Deanna Kurz, Diane K King, Tim Woolley, Andrew J Faber, Paul A Estabrooks, Lisa Strycker, Deborah Toobert, Jennifer Dickman

**Affiliations:** ^4^Oregon Research InstituteEugene, ORUnited States; ^3^Virginia Polytechnic Institute and State UniversityBlacksburg, VAUnited States; ^2^Intervision MediaEugene, ORUnited States; ^1^Institute for Health ResearchKaiser PermanenteDenver, COUnited States

**Keywords:** Engagement, Internet, diabetes self-management, research methods, health disparities

## Abstract

**Background:**

Increased access to the Internet and the availability of efficacious eHealth interventions offer great promise for assisting adults with diabetes to change and maintain health behaviors. A key concern is whether levels of engagement in Internet programs are sufficient to promote and sustain behavior change.

**Objective:**

This paper used automated data from an ongoing Internet-based diabetes self-management intervention study to calculate various indices of website engagement. The multimedia website involved goal setting, action planning, and self-monitoring as well as offering features such as “Ask an Expert” to enhance healthy eating, physical activity, and medication adherence. We also investigated participant characteristics associated with website engagement and the relationship between website use and 4-month behavioral and health outcomes.

**Methods:**

We report on participants in a randomized controlled trial (RCT) who were randomized to receive (1) the website alone (n = 137) or (2) the website plus human support (n = 133) that included additional phone calls and group meetings. The website was available in English and Spanish and included features to enhance engagement and user experience. A number of engagement variables were calculated for each participant including number of log-ins, number of website components visited at least twice, number of days entering self-monitoring data, number of visits to the “Action Plan” section, and time on the website. Key outcomes included exercise, healthy eating, and medication adherence as well as body mass index (BMI) and biological variables related to cardiovascular disease risk.

**Results:**

Of the 270 intervention participants, the average age was 60, the average BMI was 34.9 kg/m^2^, 130 (48%) were female, and 62 (23%) self-reported Latino ethnicity. The number of participant visits to the website over 4 months ranged from 1 to 119 (mean 28 visits, median 18). Usage decreased from 70% of participants visiting at least weekly during the first 6 weeks to 47% during weeks 7 to 16. There were no significant differences between website only and website plus support conditions on most of the engagement variables. In total, 75% of participants entered self-monitoring data at least once per week. Exercise action plan pages were visited more often than medication taking and healthy eating pages (mean of 4.3 visits vs 2.8 and 2.0 respectively, *P* < .001). Spearman nonparametric correlations indicated few significant associations between patient characteristics and summary website engagement variables, and key factors such as ethnicity, baseline computer use, age, health literacy, and education were not related to use. Partial correlations indicated that engagement, especially in self-monitoring, was most consistently related to improvement in healthy eating (*r* = .20, *P* = .04) and reduction of dietary fat (*r* = -.31, *P* = .001). There was also a significant correlation between self-monitoring and improvement in exercise (*r* = .20, *P* = .033) but not with medication taking.

**Conclusions:**

Participants visited the website fairly often and used all of the theoretically important sections, but engagement decreased over 4 months. Usage rates and patterns were similar for a wide range of participants, which has encouraging implications for the potential reach of online interventions.

**Trial Registration:**

NCT00987285; http://clinicaltrials.gov/show/NCT00987285 (Archived by WebCite at http://www.webcitation.org/5vpe4RHTV)

## Introduction

There is now strong evidence that Internet-based behavior change programs can be efficacious. Promising results have been reported for several lifestyle change programs relevant to diabetes management, including healthy eating and weight management [1,2], physical activity [3], and smoking cessation [4,5]. Reviewers of interactive behavior change programs for diabetes self-management have identified more than 20 randomized trials [6,7]. Many of these studies have been conducted in primary care or health system settings and have targeted high-risk individuals, including those who had low health literacy or low income or who were medically underserved [6]. The results of these trials have generally been positive.

When combined with information that the “digital divide” is shrinking in at least some respects [8], this is good news for patients with diabetes. Given the recent dramatic increases in diabetes prevalence [9] and the general reluctance of patients with diabetes to attend diabetes classes and group sessions [10,11], Internet-based and other eHealth approaches to diabetes self-management education (DSME) have great potential.

Increased access to the Internet, especially among older adults [8], and the availability of efficacious, interactive DSME programs are encouraging developments. Remaining challenges, however, are the generally low level of engagement and high attrition [12] in Internet programs [13]. Although the relationship between level of engagement and outcomes of Internet programs is unclear [5], most program developers believe that a threshold level of involvement is necessary to obtain benefit. There are also relatively few investigations of patient psychosocial characteristics associated with engagement in Internet programs. There is a substantial literature on the relationship of factors such as depression, self-efficacy, and readiness to change with engagement in DSME in general, but more data are needed on generalization of these findings to Web-based interventions.

The best methods for defining and measuring website engagement are a subject for ongoing debate [14]. Some studies have reported number of website visits; others, time spent on a site; and still others, number of components used [5,14]. In their recent review, Danaher and Seeley [14] concluded that no single, universally accepted measure of engagement exists, and they encouraged more research in this area. Given the continuing health disparities among patients with diabetes [15], investigations of patient characteristics associated with engagement are also needed. This is a complex area as different patient characteristics may be associated with participation/nonparticipation than for engagement once a person has joined a program. Different patient characteristics may also be associated with retention, level of improvement in results [13], and with level of use of different components of Web-based programs.

In this paper, we present data about program engagement from an ongoing Internet-based multimedia DSME intervention study. The intervention program was designed to address 3 self-management behaviors for adults with diabetes: medication adherence, exercise, and food choices. The website offered a number of interactive and multimedia features to enhance engagement. These included user choice of language (Spanish or English), optional audio voice-overs, choice in setting behavioral goals, and choice among various features. The site also included a changing variety of practical and fun features to keep the user experience fresh, such as rotating quiz questions and motivational tips. Significant new content at 6 weeks provided interventions more specifically tailored for each individual on the 3 primary self-management behaviors. The program integrated a variety of media, including video, still pictures, animation, and audio-narrated action plan development/refinement. Action plans listed the specific goal the patient had along with individually tailored reasons for working on this goal, barriers to be on the lookout for, and strategies to overcome these barriers. Also integrated were user forums, graphical displays of self-monitoring and laboratory test results, and prompts to return to the site via both email and interactive voice response phone calls.

The primary purpose of this paper was to report on (1) the overall rate of use of the My Path/Mi Camino diabetes self-management website among a heterogeneous sample of adults with type 2 diabetes; (2) the components of the website that were used most and least often; (3) which of a number of participant characteristics, including health literacy, ethnicity, baseline level of computer use, and medical risk factors, were associated with greater engagement with the website; and (4) the relations between different measures of engagement and 4-month outcomes.

## Methods

### Design Overview

A 3-arm, patient-level randomized practical effectiveness trial [16, 17] was employed to evaluate the impact of 2 interactive, multimedia, diabetes self-management programs, relative to “enhanced” usual care. The 2 Internet-based interventions were (1) a self-administered, computer-assisted self-management condition based on social-ecological theory and the “5 A’s” (assess, advise, agree, assist, and arrange) self-management model [18] and (2) the computer-assisted self-management program with the addition of social support from the health care team and peer group meetings. These study conditions were compared with an enhanced usual care intervention that provided health risk appraisal feedback and recommended preventive care behaviors but did not include the hypothesized key intervention processes of goal setting, barriers identification, problem solving, or social-environmental support. The remainder of this paper deals only with the 270 intervention participants.

### Recruitment

The study was conducted in primary care clinics within Kaiser Permanente Colorado (KPCO). Utilizing KPCO’s electronic prevention and disease population management system, HealthTrac, and the associated electronic medical record (EMR) system, HealthConnect, adults with type 2 diabetes were identified from 5 of the 14 KPCO primary care medical offices. Clinics were selected based on variability in size, location, and socioeconomic status of surrounding neighborhoods, and to maximize percentage of Latino patients to enhance generalizability and evaluate impacts across subgroups. Recruitment procedures are described in detail in Glasgow et al [19]. In brief, 37.9% of patients with type 2 diabetes that we contacted and who were assumed to be eligible, completed baseline assessments. Compared with those who declined, the 270 participants were likely to be younger, less likely to be Latino, had higher incomes, were much more likely to have completed postsecondary education (79% vs 53.5%), much less like to smoke (11.8% vs 19.2%, and had lower systolic blood pressure. Participants were reimbursed $25 for follow-up assessment.

Eligibility criteria included a diagnosis of type 2 diabetes made at least 1 year prior to contact, body mass index (BMI) of 25 kg/m^2^ or greater, and at least 1 other risk factor for heart disease (ie, hypertension, low-density lipoprotein [LDL] > 100 or on a lipid-lowering agent, hemoglobin A_1c_ > 7%, or being a current smoker). In addition, participants were considered eligible if they were between 25 and 75 years of age, lived independently with access to a telephone and at least biweekly access to the Internet, were able to read and write in English or Spanish, and were able to perform mild to moderate physical activity.

Demographic data were collected during the recruitment phone call. Participants were read categories from which to choose, and race and ethnicity were self-reported. Survey data, as well as height and weight, were completed via a written questionnaire collected during the baseline study visit, after informed consent and data use authorization agreements were signed. Immediately following completion of baseline surveys, participants were randomized via computer program to 1 of the 3 conditions. Information from patients’ medical records and website use data were captured electronically. The rest of this paper concerns data from the intervention conditions only.

### Interventions

The interventions were guided by a behavioral systems approach to diabetes self- management [20-23] that applies validated behavior change principles at patient, health care provider, and social-environmental levels. This strategy draws on the pioneering work of Bandura on social-cognitive theory and self-efficacy and application of social-ecologic approaches to health issues. The interventions were available in the participant’s choice of English or Spanish and were based on refinements for the Internet of interactive diabetes self-management programs found effective in our prior research [24,25].

### Computer-Assisted Self-management

**Figure 1:**
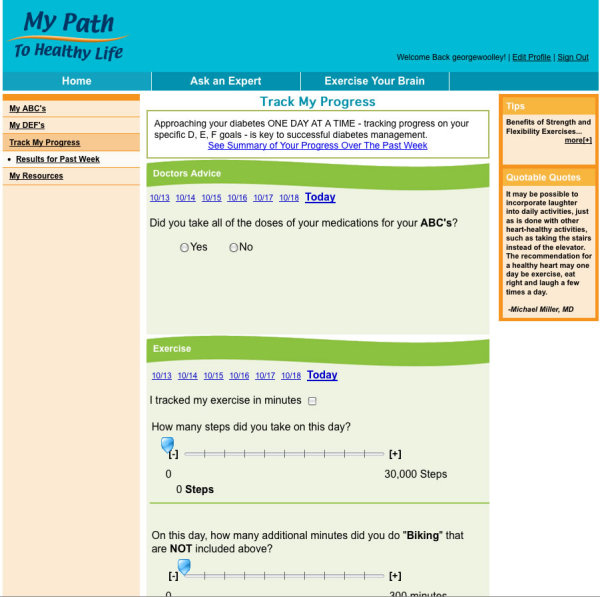
Track my progress

Participants randomized into the computer-assisted self-management condition were given access to an Internet-based website called “My Path to Healthy Life” (“My Path,” for short) in English and “Mi Camino a la Vida Sana” (or “Mi Camino”) in Spanish, which was developed in collaboration with and managed by InterVision Media, a technology company based in Eugene, Oregon. At the first visit, participants watched a short video introduction to the program narrated by coinvestigator Diego Osuna, MD, that emphasized that diabetes self-care encompassed more than sugar and blood glucose control. Dr Osuna reviewed the importance of controlling the ABCs (hemoglobin *A*_1c_, *b*lood pressure, and *c*holesterol) of diabetes by self-managing one’s “DEFs” (*d*octor’s advice regarding medication adherence, *e*xercise, and *f*ood choices). Participants were tutored in website log-in, navigation, and usage by the attending research staff member. Participants were then asked to select initial, easily achievable goals to enhance self-efficacy [7] in each of 3 areas: medication adherence, exercise, and food choices. The initial medication adherence goal involved taking doctor-prescribed diabetes, blood pressure, and cholesterol medications “the right way every day.” For exercise, participants were asked to set an initial goal of keeping track of how many steps or minutes they walked every day (pedometers were provided). Finally, for the initial dietary goal, participants were asked to eliminate their choice of fast foods, fried foods, or sugar-sweetened beverages. Participants recorded their progress on these 3 daily goals using the tracking section of the website (Figure 1) at least weekly, or alternatively, users could enter data via the interactive voice response (IVR) phone system. Participants received immediate feedback on success or struggles in tracking and meeting their goals over the past 7 days through motivational messages via both Web and IVR modalities.

During the first study visit, the research assistant briefly showed the participant each of the sections of the My Path/Mi Camino website, which included a graphical display of the patient’s hemoglobin A_1c_, blood pressure, and cholesterol results in the “My ABCs” section; a moderated forum called “Ask an Expert” where users could post or view questions to staff experts; and “My Resources,” which consisted of resources (eg, Web links, healthful recipes, and printable handouts) for diabetes self-management and healthy lifestyles. The website also contained several features designed to enhance user engagement, such as rotating quiz questions and motivational tips.

**Figure 2:**
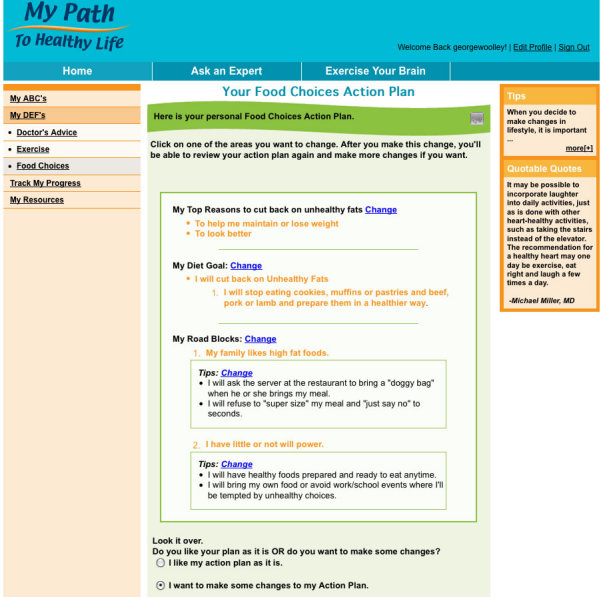
Your food choices action plan

After 6 weeks, participants were instructed to return to My Path/Mi Camino and further tailor their 3 self-management goals by creating action plans. To create action plans, participants were asked to identify motivating factors for goal attainment from a list on the screen; the option to “write your own” was also available. The medication adherence goal remained unchanged from the baseline goal to continue taking doctor-prescribed medications. For the exercise action plan, participants were shown graphs comparing their average physical activity level to national recommendations. Then, to increase their number of daily steps (or minutes of moderate exercise per day), users were asked to select 2 specific activities from a list of common activities (eg, gardening, jogging, bicycling, and walking); the “write your own” option also was available. For the food choices action plan, participants answered questions about fruit, vegetable, and fat intake, and used immediate, on-screen feedback to choose either to increase their daily number of servings of fruits and vegetables or to decrease their consumption of unhealthy fats and adhere to recommended portion sizes. To complete the problem-solving-based action-planning sequence [26] for each of the 3 areas, participants identified 2 likely barriers to achieving each of the goals they had selected and then chose from a list of strategies to overcome those barriers. Each user’s action plan (Figure 2) was stored on the website for easy reference and/or revision, and his or her EMR was updated to indicate participation in the study and include his/her action plan.

In addition to the website, computer-assisted self-management participants received periodic prompting using IVR, a computer-based telephone system that initiates outbound calls, receives inbound calls, provides information to users, and collects data from users. Study participants received a welcome call 3 days after enrolling in the study. At 6 weeks after enrollment, the action plan feature was added to the website and participants were prompted by IVR and email to revise their D, E, and F goals through completing the action plans in each of the 3 areas. They were reminded again after 5, 15, and 25 days if action plans had not been completed. The IVR also prompted participants to return to the website to track their progress after 6 days of missed tracking. Participants were first reminded via email, and then 3 IVR contact attempts were made per day for 3 consecutive days at 5, 15, and 25 days after the initial email.

### Computer-Assisted Self-management Plus Social Support

Participants randomized to the computer-assisted self-management plus social support group received all aspects of the website intervention with the addition of follow-up calls and were invited to attend group visits with other participants in the same study condition. The 2 extra follow-up calls occurred 2 and 8 weeks after the initial visit. The first follow-up call was completed by the same study staff member who conducted the initial visit; its purpose was to answer any study-related questions and troubleshoot problems with the website or initial self-management goals. The second call was completed by a KPCO diabetes care manager to discuss the participant’s action plans. These semistructured calls lasted approximately 10 minutes. In addition, 1 group session was held prior to the 4-month assessment. The session focused on healthy eating. Led by a bilingual KPCO nutritionist, the meeting included information on healthy restaurant eating behaviors and grocery shopping tips.

### Measures

#### Patient Characteristics

Demographic variables included self-reported age, gender, race, Latino ethnicity (yes vs no), household income, and education. Self-efficacy was assessed with Lorig’s 8-item Diabetes Self-Efficacy Scale [27]. In addition, 6 similarly constructed self-efficacy items recommended by Bandura [28] were added to measure confidence regarding taking diabetes medications, exercising, and limiting high-fat foods. Self-efficacy subscales were calculated for healthy eating, physical activity, and medication taking. Problem-solving skill was assessed with the Positive Transfer of Past Experience from the Diabetes Problem Solving Scale of Hill-Briggs [29].

#### Health Literacy and Baseline Computer Use

During the recruitment call, all participants were assessed for health literacy using the 3 items identified as most sensitive from the widely used instrument to assess health literacy, the Short Test of Functional Health Literacy (STOFHL) [13]. Extent of computer use was assessed by a single question asking how many hours per week on average the respondent spends on a computer.

#### Eating Patterns

Fat intake was measured by the National Cancer Institute Percent Energy from Fat Screener (PFAT) [30], which assesses intake of 15 foods selected to optimally predict percent energy from fat. Eating behaviors were assessed using the 7-item dietary assessment, Starting the Conversation instrument [31].

#### Physical Activity

The 28 physical activity items from the Community Healthy Activities Model Program for Seniors (CHAMPS) questionnaire [32] were used to measure self-reported physical activity, calculated as total weekly caloric expenditure in all physical activity. In studies of older adults that compared interviewer data and activity logs with the CHAMPS, the latter demonstrated good construct validity, stability, and sensitivity to change [32, 33] and has also been previously validated with estimated maximal oxygen consumption [34].

#### Medication Adherence

Adherence to diabetes, blood pressure, and cholesterol medications was assessed through the medication-taking items of the Hill-Bone Compliance Scale [35] that determines how often and why respondents missed taking medications.

#### Biological Outcomes

Biologic variables included: BMI, hemoglobin A_1c_, total cholesterol, LDL cholesterol, high-density lipoprotein (HDL) cholesterol, systolic blood pressure, smoking status (yes/no and number of cigarettes per day for smokers), and diabetes medication regimen. Hemoglobin A_1c_ was measured on a Bio-Rad Variant II Turbo liquid by high-pressure liquid chromatography. BMI (kg/m^2^) was obtained from electronic medical records as well as height and weight measurements obtained during in-person assessments. Lipids were assayed on a modular chemistry analyzer from Roche Diagnostics. The total cholesterol test was a serum test that first removed the cholesterol from its esters and then measured the free concentration biochemically through a modified version of the Abell Kendal method. LDL cholesterol was calculated unless the triglyceride was greater than 399 mg/dL, in which case it was measured directly with Roche assay on the modular chemistry analyzer. The UKPDS (United Kingdom Prospective Diabetes Study) 10-year heart disease risk score [36] was calculated for all study participants. The formula predicts occurrence of new heart disease in people with type 2 diabetes and incorporates hemoglobin A_1c_, systolic blood pressure, and lipid levels along with age, sex, race, smoking status, and time since diabetes diagnosis [37].

#### Website Usage

Website use was calculated from automated data in multiple ways due to the nonnormal distributions of several of the use statistics. Use of various sections was indexed by presentation of mean number of visits, median number of visits, and the percentage of patients who visited each section of the website at least twice (eg, “Tracking My Progress,” “Action Planning,” “My ABCs,” “Ask an Expert,” and “Resources”). The number of action plans created (out of a possible 3) also was computed. For the self-monitoring activities, we calculated the percent of days for which tracking data were entered on the website for each of the 3 target behaviors. Time spent on the site for each visit was calculated as follows (excluding page view times exceeding 30 minutes): total time on site per visit = (last page visit time – log-in time) +(last page visit time – log-in time)/(n – 1 total pages visited). Visit times were summed to reflect total time engaged in the site across the intervention period. Key summary engagement variables were: total number of log-ins per participant and the number of website components visited at least twice (range 0 to 5).

### Analyses

All survey data were entered and verified, and scores were calculated for multiple-item instruments according to previously established procedures (eg, dietary fat intake, UKPDS risk of coronary heart disease). EMR data were merged with website use and survey data for analysis. Descriptive statistics were computed for all variables to determine the nature of the data and to test for normality assumptions.

Chi-square and *t* tests were used to compare baseline participant characteristics between the website and website plus human support conditions and to test for treatment group differences in website usage.

To investigate potential patient characteristics associated with website use, Spearman nonparametric correlations were calculated between participant characteristics and 5 summary variables characterizing website usage.

To identify potential website engagement factors associated with outcomes, partial correlations were computed between the website use variables and the key behavioral outcomes of healthy eating, physical activity, and medication taking, and the key biological outcomes of BMI, hemoglobin A_1c_, and the UKPDS heart-disease risk measure. These partial correlations controlled for treatment condition, baseline scores on the relevant outcome measure, and participant characteristics that were significantly related to outcomes (gender, age, and ethnicity).

## Results

### Participant Characteristics

As can be seen in Table 1, participants were fairly typical of patients with type 2 diabetes in general. The average age of the 270 participants was 60 years, 48% (130/270) were female, their mean BMI was 34.9 kg/m^2^ (classified as obese), and they had an average baseline hemoglobin A_1c_ of 8.2%. As with type 2 diabetes nationally, this sample contained a higher proportion of Latino, African American, and Native American individuals than were in the general KPCO membership. The majority of participants reported using a computer 9 or more hours per week, although 18% (49/270) reported using computers 2½ or fewer hours per week. Income was highly variable with 45% (122/270) of participants reporting annual family incomes less than US $50,000 and 18% (49/270) reporting annual family incomes over $90,000. There were no significant between-condition differences of participant characteristics at baseline.

**Table 1:** Baseline characteristics of participants (n = 270)

		All	Website n = 137	Website Plus Human Support n = 133	*P* Value
Age (years), mean (SD)		57.8 (9.3)	58.0 (0.4)	57.6 (9.3)	.697
% Female		48.1%	45.3%	51.1%	.334
**Race**					.891
	American Indian/Alaska Native, %	4.2%	5.1%	3.0%	
	Asian, %	1.5%	1.5%	1.5%	
	Black or African-American, %	18.1%	16.8%	19.5%	
	White, %	67.4%	69.3%	65.4%	
	No information/other, %	8.9%	7.3%	10.5%	
Latino ethnicity (yes vs no)		22.3%	24.8%	19.8%	.333
**Income**					.965
	Less than US $49,999, %	44.8%	45.6%	44.4%	
	US $50,000 to US $89,999, %	30.7%	30.0%	31.6%	
	US $90,000 or more, %	18.5%	18.2%	18.8%	
	No information, %	5.9%	6.6%	5.3%	
High School or less education, %		20.4%	18.4%	22.6%	.396
Health literacy score, mean (SD)		4.8 (0.4)	4.8 (0.5)	4.8 (0.4)	.314
**Computer use, %**					.813
	Never to 2.5 hrs per week, %	17.8%	16.8%	18.8%	
	3 to 6.5 hrs per week, %	15.6%	19.0%	12.0%	
	7 to 8.5 hrs per week, %	7.0%	5.8%	8.3%	
	9 or more hrs per week, %	59.6%	54.4%	60.9%	
Smokes cigarettes, % (yes/no)		11.1%	9.5%	12.8%	.389
Body mass index (kg/m^2^), mean (SD)		34.9 (6.6)	34.6 (6.3)	35.2 (6.9)	.479
Systolic blood pressure (mm Hg), mean (SD)		130.3 (15.7)	130.7 (16.6)	129.9 (14.7)	.680
Diastolic blood pressure (mm Hg), mean (SD)		77.7 (10.4)	77.9 (9.8)	77.5 (11.0)	.762
Hemoglobin A_1c_, mean (SD)		8.2 (1.8)	8.1 (1.9)	8.3 (1.7)	.395

### Website Use

As can be seen in Table 2, participants demonstrated large variability in website usage over the 4 months of data collection, ranging from 1 to 119 website visits (mean 28 visits, median 18). Usage decreased over time, with 70% of those randomized visiting at least weekly during the first 6 weeks and 47% during weeks 7 to 16. More detailed analyses by week revealed a gradual decrease in the frequency of use over the 16 weeks, with a modest spike around the time of the 6-week prompts and addition of the action planning component. Total time spent on the website during the 4 months averaged a little over 3 hours, or about 7 minutes per visit, with a median of 152 minutes total time on the site. There were no significant differences between website alone and website plus human support conditions on any of the overall use variables.

The “Track My Progress” self-monitoring section, as anticipated, was the most frequently visited part of the website. More than 75% (208/270) of all participants used the tracking feature an average of at least once per week. Across the 4-month period, percent of days tracked ranged from 50% to 58% (mean and median) for each of the 3 targeted behaviors. Participants with access to the website plus human support entered tracking data for medication adherence more frequently than did participants with access to the website alone (*P* = .02), but this was not true for the other 2 behaviors. In general, participants who entered data for 1 behavior also entered data for the other behaviors at that time.

The “Action Plan” section of the website was considered by the program designers to be the other most important component. Participants completed an average of 1.7 of the 3 action plans, with about two-thirds completing exercise and healthy eating plans, and slightly less than half completing medication-taking plans. As can be seen in Table 2, users visited the exercise section of the “Action Plan” area significantly more often than for the other 2 behaviors (mean of 4.3 visits vs. 2.8 and 2.0 for medication and healthy eating, respectively, *P* < .001).

All pages of the website were used relatively often. The “My ABCs” page that graphically displayed the user’s lab results was the third most frequently visited page after the “Track My Progress” and “Action Plan” pages, followed by the “Resources” and “Ask an Expert” pages. The various pages were each visited at least twice by 43% or more of the users, and 19% of users visited all pages at least twice. Participants allocated to website plus human support visited most of the pages slightly more often than participants with access to the website alone, which cumulatively resulted in a composite website section use summary score that indicated significantly greater overall usage among participants in the website plus human support condition (*P* = .04).

**Table 2:** Website usage (0 to 4 months) overall and by treatment condition

Variable and Measure				All n = 270	Website n = 137	Website Plus Human Support n = 133	*P* Value
**Total number of visits to website**							
		Mean (SD)		27.8 (26.6)	27.9 (31.2)	27.7 (25.9)	.936
		Median		18	15	20	
		Range		1-119	1-119	1-112	
**Participants that visited at least weekly**							
		From 0 to 6 weeks, %		70%	66%	74%	.149
		From 6 weeks to 4 months, %		47%	44%	51%	.228
**Total time spent on website (minutes)**							
		Mean (SD)		190 (174)	183 (177)	196 (171)	.537
		Median		152	143	165	
		Range		9-1008	11-882	9-1008	
**Self-monitoring (% days tracked)**							
		**Medications**					
			Mean	50%	43%	57%	.017
			Median	53%	39%	69%	
			Range	1% - 100%	1% -100%	1% - 100%	
		**Physical activity**					
			Mean	53%	50	55	.260
			Median	58%	51	61	
			Range	0% - 100%	0% - 100%	0% - 100%	
		**Healthy eating**					
			Mean	53	51	55	.402
			Median	58	52	62	
			Range	0% - 100%	0% - 100%	1% - 100%	
**Self-monitoring (% of participants that tracked an average of at least once per week)**							
		Medications, %		78%	76%	80%	.459
		Physical activity, %		77%	74%	79%	.381
		Healthy eating, %		78%	76%	80%	.459
**Total number of action plans completed excluding those ineligible for medication adherence action planning**							
		Mean (SD)		1.7 (1.4)	1.5 (1.4)	1.9 (1.4)	.083
		Median		3	2	3	
		Range		0-3	0-3	0-3	
**Action plan visits**							
		**Medications (among eligible participants)**					
			Mean (SD)	2.8 (5.1)	2.4 (4.3)	3.3 (5.8)	.140
			Median	3	0	3	
			Range	0-41	0-21	0-41	

		**Physical activity**					
			Mean (SD)	4.3 (5.7)	4.0 (4.6)	4.7 (6.7)	.321
			Median	3	3	3	
			Range	0-46	0-25	0-46	
		**Healthy eating**					
			Mean (SD)	2.0 (2.0)	1.9 (1.9)	2.2 (2.1)	.276
			Median	2	2	2	
			Range	0-12	0-25	0-12	
		**Ask an expert visits**					
			Mean (SD)	2.7 (4.7)	2.5 (4.6)	2.9 (4.8)	.438
			Median	1	1	1	
			Range	0-45	0-45	0-43	
		**ABC visits**					
			Mean (SD)	5.3 (6.0)	5.1 (5.3)	5.5 (6.6)	.574
			Median	4	3	4	
			Range	1-54	1-36	1-54	
		**Resources visits**					
			Mean (SD)	4.9 (5.6)	4.6 (4.3)	5.2 (6.6)	.410
			Median	3	3	3	
			Range	0-54	0-11	1-54	
**Progress summary page visits**							
		Mean (SD)		19.4 (24.2)	19.5 (25.5)	19.3 (22.8)	.957
		Median		10	10	10	
		Range		0-145	0-145	0-124	
		**Composite score**					
			Mean (SD)	3.3 (1.6)	3.1 (1.7)	3.6 (1.5)	.041
			Median	4	4	4	
			Range	0-5	0-5	0-5	

### Correlates of Website Use

Of the large number of nonparametric correlation coefficients computed between patient characteristics and website use, none was larger than .19 and there was no consistent pattern of relationships. None of the clinical variables in Table 1 was significantly related to any of the summary website use variables nor were ethnicity, education, health literacy, or baseline level of self-efficacy, problem-solving skill, or computer use. These results suggest that a wide range of participants, including those at highest risk, were equally able and likely to use the site.

### Relationship Between Use and Outcomes

The final issue analyzed was the relationship between website use, using the same 5 summary usage variables as above and improvement in key outcome variables from baseline to the 4-month assessment. Table 3 presents partial correlations between the website use variables and the key 4-month behavioral and biological outcomes controlling for treatment condition, baseline scores on the relevant outcome measure, and participant characteristics that were significantly related to outcomes (gender, age, and ethnicity). Website use was most consistently related to the dietary measures. These moderate-sized correlations indicate that greater use of the website, and especially engagement in self-monitoring, was related to greater improvement in eating patterns. There was also a significant relation between self-monitoring and improvement in physical activity but not with medication adherence. None of the biological outcomes was significantly associated with the engagement measures.

**Table 3:** Behavioral and clinical correlates of website usage (n = 167 for the 4 behavioral measures; n = approximately 157 for the 3 biological measures; slight variation by measure in that n = 110 for medication because not all on diabetes medications)

		Healthy Eating *r*	Fat Intake *r*	Physical Activity *r*	Medication Adherence *r*	BMI *r*	Hemoglobin A_1c_ *r*	10-year UKPDS *r*
**Self-monitoring**								
	Medication taking (n = 110)	.31^b^	-.28 ^b^	.17	.16	-.03	.04	-.15
	Physical activity	.29 ^b^	-.20 ^a^	.20 ^a^	.16	-.05	-.03	-.17
	Healthy eating	.29 ^b^	-.20 ^a^	.22 ^a^	.15	-.05	-.04	-.16
Number of action plans		.21 ^a^	-.20 ^a^	.05	.15	.01	.05	-.15
Total number of visits		.20 ^a^	-.11	.14	.17	-.09	-.07	-.07
Total time (minutes)		.37 ^c^	-.20 ^a^	.17	.15	-.09	-.03	-.15
Comprehensive Web use score (n = 123)		.20^a^	-.31 ^b^	.00	.11	.00	.06	-.14

^a^ *P* < .05

^b^ *P* < .01

^c^ *P* < .001

## Discussion

Our primary goal was to report on the level of use of our Internet DSME and the site components used most and least often. Overall, and compared with a number of prior Internet lifestyle change programs [5,17], the My Path/Mi Camino website was well used. During the initial weeks of the program, the vast majority of users logged into the site at least once per week, the minimum expectation of users, and some users visited the site daily. As has been reported previously in a study of another Web-based diabetes self-management program [38], use of the My Path/Mi Camino site decreased over time but was still moderately high by 4 months. Website utilization varied widely. By 4 months, some users had stopped or visited only sporadically; many users visited the site approximately weekly (usually to enter self-monitoring data), and some continued to use the site almost daily. Continued usage by the latter groups may have been due to a combination of the design features of the website to promote “stickiness,” including a high degree of interactivity and choice, voice-overs, a variety of visual and auditory displays, frequent updates and changes, the ability for users to further tailor the recommendations and strategies by writing their own alternatives, and feedback on both behavior-change targets and laboratory results, along with prompts and reminders to keep users involved.

Usage rates were similar on most engagement measures in the website and the website plus human support conditions, with the website plus human support condition producing slightly higher rates that occasionally reached statistical significance (eg, on the composite section use score and the number of days self-monitoring data were entered on medication use, but not on the other 2 target behaviors). Given moderately high use in the website alone condition, it may be that more frequent or intensive added support or contacts are needed to substantially increase usage above this level. Increased linkage to the primary care team, such as individualized emails to participants from health counselors as used successfully in weight loss website interventions by Tate and colleagues [2], might enhance usage but would also add costs and staff time.

All of the key features of the My Path/Mi Camino site were utilized. As expected, a high percentage of participants fairly regularly reported self-monitoring data (78% at least weekly), but use of the action plan pages was more infrequent than expected. In particular, the healthy eating action planning section use was low. This may have been because of navigational difficulties in updating or modifying dietary goals and strategies compared with the ease of revising the more frequently visited physical activity action plans. Our data suggest that older patients with diabetes can simultaneously monitor multiple health behaviors [39].

After the “Track My Progress” section, the next most frequently visited section of the My Path/Mi Camino website was the “ABCs” section that presented graphical displays of laboratory results. Given these results and the availability of the “Ask an Expert” section, which was used moderately often, especially initially, users may have found the website a useful extension of their diabetes care.

A secondary goal was to evaluate the association between a variety of patient characteristics and website use. Even given heterogeneity in both participant characteristics and website engagement measures, the associations between these variables were low. Though a large number of correlations were computed, none reached the Spearman *r* = .20 level and thus are not considered to be clinically important; the few patient characteristics that were significantly associated with 1 or 2 engagement measures did not replicate across other engagement measures. On one hand, the general lack of patient characteristics predictive of engagement fails to suggest how to improve the website. On the other hand, our results indicate that a wide range of persons with a variety of education, age, income levels, ethnic backgrounds, sociodemographic, psychosocial, and clinical characteristics were able to use the website. It was especially encouraging that participants who were older or of Latino ethnicity as well as those with a higher risk of diabetes complications, or who had moderate to lower health literacy, or who had little baseline computer use were as engaged with the website as other participants. It may have been that efforts made during website development to address issues of literacy, cultural appropriateness, patient-centeredness, and personal choice and to maximize initial success served to make the program engaging to a broad variety of patients. Given the emphasis on self-monitoring and graphical feedback, it would have been helpful to have collected measures of health numeracy as well as general health literacy.

Several of the engagement measures (self-monitoring measures, action plan engagement, total number of visits, total time on the site, and the composite section use index) were moderately related to improvement in healthful eating behaviors over 4 months. Website engagement was not, however, related to improvement in the other target behaviors or in biological outcomes.

We created a number of engagement measures that were relevant to our particular study. The pattern of results does not suggest the superiority of any particular engagement measure over the others. As measures that could be used across a variety of Internet intervention areas, we recommend (1) total visits and (2) a composite score to reflect overall use of different sections similar to that used by Strecher et al [5]. Because of the often skewed and nonnormal distribution of engagement scores, we also recommend inspection of scatter plot displays of the relationship between engagement and outcomes and investigation of dichotomous “threshold use” indices (eg, percent of participants that used the site or a section a minimum number of times believed to be required). To understand the engagement construct, additional qualitative data such as patient interviews would have been helpful.

Limitations of this report included the use of a single managed care setting and the relatively short 4-month time frame. Strengths included a large and diverse sample, inclusion of a number of patient health-disparity characteristics, digital divide issues (eg, baseline level of computer use, gender, race, and age), and the variety of engagement measures available from automated data. Future research recommendations include investigation of levels of engagement across different clinical settings and qualitatively different components of interactive programs (eg, information vs problem-solving or peer support components) with different levels of linkage to the primary care team, use of a sophisticated measure of health numeracy, as well as a more sophisticated measure of health literacy.

## Abbreviations

BMI: body mass index
CHAMPS: Community Healthy Activities Model Program for Seniors
DSME: diabetes self-management education
EMR: electronic medical record
HDL: high-density lipoprotein
IVR: interactive voice response
KPCO: Kaiser Permanente Colorado
LDL: low-density lipoprotein
PFAT: percent energy from fat
RCT: randomized controlled trial
STOFHL: Short Test of Functional Health Literacy
UKPDS: United Kingdom Prospective Diabetes Study
